# Oral lesions of viral, bacterial, and fungal diseases in children: A decision tree

**DOI:** 10.3389/fped.2022.937808

**Published:** 2022-07-25

**Authors:** Charlotte Guillouet, Margot C. Riou, Lucas T. Duong, Muriel de La Dure-Molla, Benjamin P. J. Fournier

**Affiliations:** ^1^Laboratory of Embryology and Genetics of Human Malformations, INSERM UMR 1163, Institut Imagine, Université Paris Cité, Paris, France; ^2^Université Paris Cité, Dental Faculty, Department of Pediatric Dentistry, Paris, France; ^3^AP-HP, Rothschild Hospital (ORARES), Dental Department, Reference Center for Oral and Dental Rare Diseases, Paris, France; ^4^Centre de Recherche des Cordeliers, Université Paris Cité, Sorbonne Université, INSERM UMRS 1138, Molecular Oral Pathophysiology, Paris, France; ^5^AP-HP, Charles Foix Hospital, Oral Surgery Department, Paris, France; ^6^Gustave Roussy, Paris-Saclay University, Department of Head and Neck Surgical Oncology, Villejuif, France; ^7^Bases Moléculaires et Physiopathologiques des Ostéochondrodysplasies, INSERM UMR 1163, Institut Imagine, Université Paris Cité, Paris, France; ^8^Université Paris Cité, Dental Faculty, Department of Oral Biology, Paris, France

**Keywords:** decision trees, communicable diseases, oral manifestations, mouth mucosa, oral medicine, pediatric dentistry

## Abstract

Oral mucosal lesions are common in the pediatric population and, apart from traumatic and tumoral etiologies, they can be symptoms of viral, bacterial, fungal or parasitic diseases. Yet, pediatricians and pediatric dentists find it challenging to reach a diagnosis and provide appropriate care when facing lesions of the masticatory or lining mucosa, of the hard or soft palate, of the tongue or salivary glands. Here, we propose a decision tree for the diagnosis of the most frequent viral, bacterial, and fungal diseases starting from their oral lesions in children. By first focusing on describing the elementary lesion itself before its localization and characteristics, it aims to guide the practitioner toward the diagnosis and any necessary complementary exams. To generate this tool, we conducted a literature review of the childhood viral, bacterial, fungal and parasitic diseases with oral mucosal symptoms. For each of the 42 reported diagnoses−20 viral, 9 bacterial, 5 fungal, and 8 parasitic—we collected the infection mechanism and agent(s), the oral lesions and their description, the associated systemic signs and the incidence/prevalence. In fine, our decision tree indexes the 28 diseases for which epidemiological data was available, mainly in Europe and the United States.

## Introduction

The incidence of oral mucosal lesions in children of any age is not precisely known but these lesions are frequent (1). They can be detected incidentally during medical or dental examinations or motivate emergency appointments if pain is associated or if the patient's general condition is altered. The lesions' characteristics allow the practitioner to reject traumatic or tumoral etiologies and consider an infectious etiology. In that case, the lesions can be the prodrome or symptom of a systemic disease or sign a local infection (2). This outlines the importance of a precise terminology to reach the diagnosis and adopt the adequate therapeutic strategy. The basic descriptive terms (e.g., vesicle) used in this work follow the 2016 International League of Dermatological Societies' revised glossary (3) (Supplementary Table 1 for detail).

While some infections like herpetic stomatitis (4) are easily recognizable in children, others lead to more subtle clinical features that can be challenging for pediatricians and pediatric dentists to diagnose. Yet, to our knowledge, there is no extensive review of the oral mucosal lesions of viral, bacterial, fungal and parasitic diseases in children. Therefore, we conducted a literature review of these childhood communicable diseases with oral mucosal symptoms and indexed these lesions. We assembled the relevant clinical data from our review into a decision tree for the diagnosis of the most frequent viral, bacterial, and fungal diseases starting from their oral lesions in children.

## Background for the development of the decision tree

### Research strategy of the review

We conducted a review of the literature in PubMed database of the childhood viral, bacterial, fungal and parasitic diseases with oral mucosal symptoms. To do so, we performed a literature analysis to identify the best documented diseases. Following PRISMA guidelines and flowchart, we developed a research equation which retrieved 2,380 references (5). First, we focused on reviews and we included 23 articles by title/abstract and full-text screening. Second, we refined our research equation by excluding the 25 diseases whose oral lesions were well described in the initial 23 reviews. This retrieved 1,369 references of which 62 were included (Supplementary Data 1; Supplementary Figure 1 for research equations and articles selection process).

We included all types of articles (case reports, case series, and reviews) in English or in French, describing oral lesions of viral, bacterial, fungal or parasitic diseases in human subjects aged 0–18 years. We excluded articles that did not describe oral lesions of the disease or did not describe the etiology of the oral lesion and articles that did not meet the inclusion criteria. We extracted data from the 85 articles included: the infection mechanism and agent(s), the oral lesion(s) and their description, the associated systemic signs, any necessary complementary exams and the incidence/prevalence of the disease. We used the Oxford Center for Evidence Based Medicine classification (6) to assess each article's level of evidence.

### Viral, bacterial, fungal, and parasitic diseases with associated oral lesions in children

Our review reported 42 diagnoses of diseases with oral lesions, presenting as 8 different types of lesions: macule, papule, plaque, vesicle, bulla, nodule, erosion, ulcer. Twenty etiologies of these diseases were viral: herpetic stomatitis and herpes labialis, chickenpox and zona, infectious mononucleosis, cytomegalovirus infection, roseola, Kaposi sarcoma, papilloma, focal epithelial hyperplasia, condyloma acuminata, molluscum contagiosum, erythema infectiosum, herpangina, hand-foot-and-mouth disease, dengue, chikungunya, measles, mumps and HIV-1 infection. Nine etiologies of these diseases were bacterial: scarlet fever, bacterial tonsillitis, actinomycosis, noma, noma neonatorum, erythema multiforme, tuberculosis, leper and syphilis. Five etiologies of these diseases were fungal: candidiasis, paracoccidioidomycosis, histoplasmosis, aspergillosis and mucormycosis. Eight etiologies of these diseases were parasitic: leishmaniasis, toxoplasmosis, ascariasis, onchocerciasis, cysticercosis, hydatid echinococcosis, schistosomiasis and myiasis.

Out of the 42 diagnoses, 6 diseases led to one or multiple papules, one disease led to macules; 7 diseases led to plaques, 7 diseases led to vesicles or bullae that secondarily led to erosions or ulcers; 7 diseases led to one or multiple nodules, 10 diseases led to one or multiple ulcers, and 5 diseases led to the tumefaction of a salivary gland. Five of the 42 diseases have pathognomonic oral signs: uvulo-palato-glossal ulcers in roseola (7), Koplik spots in measles (8), strawberry tongue in scarlet fever (9), intra-lesion sulfur granules in oral actinomycosis (10) and cauliflower-like aspect of papilloma (4). These findings show that clinically characterizing the lesions is not sufficient for the 37 other diagnoses. Complementary exams like oral swabs or biopsies may be required to confirm the positive diagnosis. This further highlights the relevance of designing a decision tree.

Of the 85 articles included, 48 were case reports or case series [Oxford evidence level 4 (6)], 13 were individual prospective or retrospective studies (level 3), 24 were reviews of the literature without systematic objectives and/or methods (level 3).

### Decision tree

**Figure 1:**
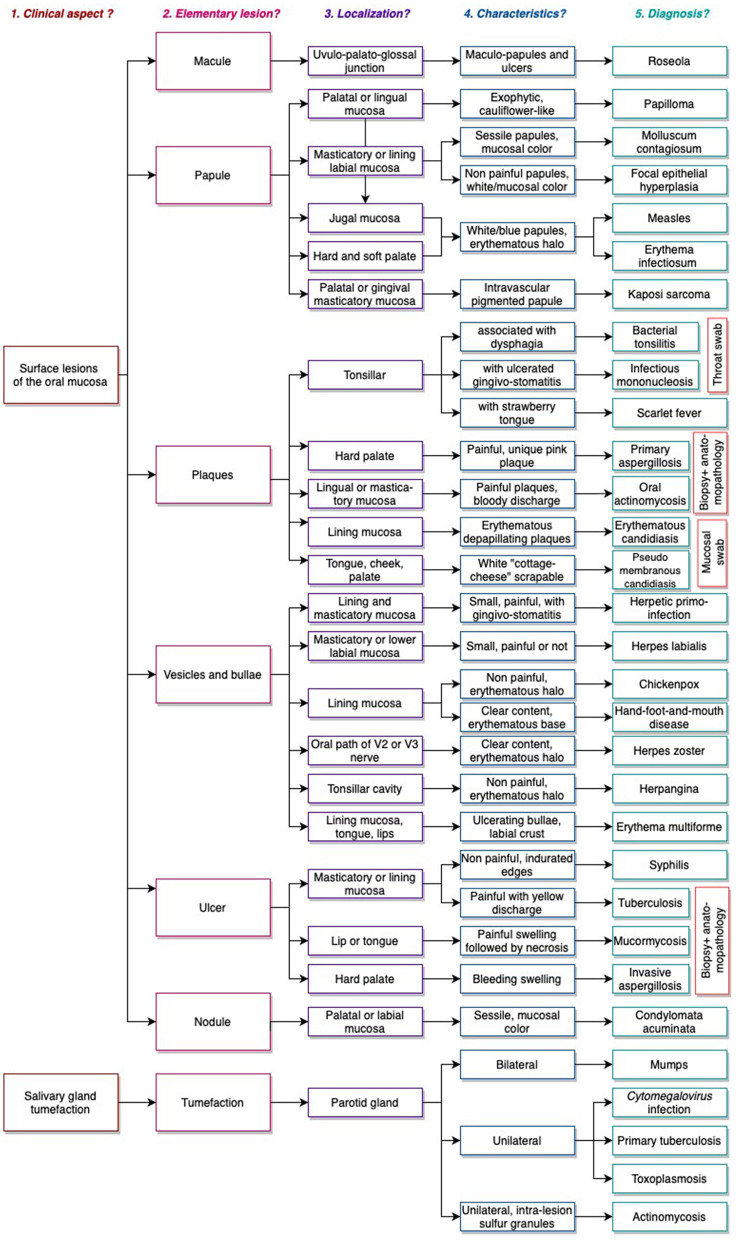
Decision tree.

To create a relevant tool associating the oral lesions with the most common diagnoses encountered by pediatricians and pediatric dentists, we decided to generate a decision tree (Figure 1). Our review found epidemiological data for 28 diagnoses, mainly in Europe and the United States (Table 1). Tropical viral fevers (chikungunya and dengue), specific bacterial diseases (leper, noma and noma neonatorum), endemic fungal diseases (paracoccidioidomycosis and histoplasmosis) and parasitic diseases were therefore excluded due to a lack of available epidemiological data.

**Table 1:** Epidemiological data in the general population for the 28 diseases included in the decision tree (HPV, human papillomavirus; /, no data available; HHV, human herpesvirus).

	**Europe**	**United States**
Condyloma acuminata, papilloma and focal epithelial hyperplasia	Oral HPV prevalence: 12.3–48.1% of immunocompetent children (11)	*/*
Mumps	6 cases/100,000 inhabitants in 2017 (12)	3,474/100,000 inhabitants in 2019 (13)
Cytomegalovirus infection	0.65% of births (14)	>1/3 children infected before 5 years old (13)
Infectious mononucleosis	50 cases/100,000 inhabitants per year (12)	/
Herpetic stomatitis and herpes labialis	HHV1 infection: endemic and global in the world (15)	
Chickenpox	8,540 cases/100 000 inhabitants in 2017 (12)	8,297 cases/100,000 inhabitants in 2019 (13)
Herpes zoster	/	1/3 of adult population (13)
Hand-foot-and-mouth disease and herpangina	/ (no mandatory notification: unknown prevalence/incidence in France and the United States)	
Molluscum contagiosum	Global endemic, 8.6% in children <16 years old in Spain (16)	/
Measles	3.9 cases/100,000 inhabitants in 2019 (12)	1,282 cases/100 000 inhabitants in 2019 (13)
Erythema infectiosum	Seroprevalence Parvovirus B19: 2–15% of children <5 and 15–60% in 5–19 years old (17)	/
HIV-1 infection	0.9/100,000 births in 2018 (18)	31,000 new cases in 2019 (>13 years old) (13)
Roseola	Seroprevalence HHV6: 100% of general population (19)	/
Actinomycosis	5 cases/100,000 inhabitants (20)	/
Tuberculosis	10 cases/100,000 inhabitants in 2020 (12)	8,916 cases/100 000 inhabitants in 2019 (13)
Scarlet fever and bacterial tonsillitis	/ (***S. pyogenes*** accounts for > 40% of strep throat in children (21)	
Syphilis	1,762 cases in 2018 (12)	38,992 new primary and secondary cases 2019 (13)
Erythema multiforme	Prevalence <1% (22)	
Mucormycosis	0.08 cases/100,000 inhabitants in 2010 (23)	*1.7 million cases a year* (13)
Primary and invasive aspergillosis	1.8 cases/100,000 inhabitants (24)	15,000 hospitalizations in 2014 (13)
Erythematous and pseudomembranous candidiasis	0.01–3.7% of children (2)	/
Toxoplasmosis	200,000–300,000 cases per year (25)	Seroprevalence: 11% of population > 6 y. old (13)

Our decision tree is based on the following diagnostic approach: (1) What is the general clinical aspect? (2) What type (macule, papule, plaque, vesicle, bulla, nodule, erosion, ulcer, tumefaction) and how many lesions are there? (3) What is its/their location (salivary gland, tongue, hard/soft palate, masticatory mucosa, lining mucosa)? (4) What are its/their characteristics? (5) What is the diagnosis?

## Discussion

The most common childhood diseases and their associated oral lesions have been studied in other reviews (4, 26, 27), but our work had an extensive aim. Reviewing the childhood viral, bacterial, fungal and parasitic diseases with oral signs allowed us to create a decision tree that may be used by any pediatrician and pediatric dentist in Europe and the United States.

We have shown that despite the number of diseases studied, their oral lesions are limited to eight different types of lesions. For example, an oral mucosal nodule can refer to a parasitic cyst (28) or to an epithelial growth in a case of condyloma acuminata (4). Given this ambiguity, basic descriptive terms (3) must be considerably strengthened by additional characteristics. This work also sheds light on the existence of oral pathognomonic signs that practitioners should be aware of, for roseola, measles, scarlet fever, oral actinomycosis and papilloma. They should also be aware of the cases for which an oral swab, a mucosal biopsy and anatomopathological examination or a specific serology is required.

As the difficulty when facing an oral mucosal lesion is to find its etiology to provide appropriate care, our decision tree should further be enriched with the oral lesions of other etiologies. A traumatic etiology should be considered when the lesion presents an edematous aspect with a fibrinous base and heals within 10 days after the removal of its cause. A tumoral etiology should be considered when the lesion presents an indurated and/or inhomogeneous aspect with an ulcerated and/or necrotic base and elevated edges, associated with lymphadenopathies and/or spontaneous bleeding. Mucosal lesions of dental infections and periodontal diseases (e.g., abscess and gingivitis), not included in the field of this work, should also be added to complete the decision tree.

To our knowledge, there is no existing equivalent decision tree. Its reproducibility and reliability must be demonstrated, which we plan on doing in the Pediatric Dentistry Department of Rothschild Hospital in Paris. Any new clinical tool must be validated but it is crucial for our decision tree because our selection of articles contains only low levels of evidence. This is clearly inherent to our field of research but also introduces a risk of bias. We must evaluate our decision tree's clinical validity in order to define its frame of use. Afterwards, its intrinsic validity (specificity and sensitivity) and extrinsic validity (positive and negative predictive values) need to be investigated in real clinical situations.

## Data availability statement

The original contributions presented in the study are included in the article/supplementary materials, further inquiries can be directed to the corresponding author.

## Author contributions

BF conceived the ideas. CG, BF, and MR collected and analyzed the data. CG, LD, and MDLDM led the writing redaction of manuscript. All authors contributed to the article and approved the submitted version.

## Funding

This work was funded by INTERFACE INSERM/APHP Contract (BF).

## Conflict of interest

The authors declare that the research was conducted in the absence of any commercial or financial relationships that could be construed as a potential conflict of interest.

## Publisher's note

All claims expressed in this article are solely those of the authors and do not necessarily represent those of their affiliated organizations, or those of the publisher, the editors and the reviewers. Any product that may be evaluated in this article, or claim that may be made by its manufacturer, is not guaranteed or endorsed by the publisher.
