# Supplementary material for: Oral lesions of viral, bacterial, and fungal diseases in children: A decision tree
Source: Front Pediatr. 2022 Jul 25;10:937808. doi: 10.3389/fped.2022.937808 (PMC9358008; doi:10.3389/fped.2022.937808)

Supplementary Material in order of appearance in main text

Supplementary Table 1. Definitions of the basic descriptive dermatological terms used in the decision tree, following the 2016 International League of Dermatological Societies' revised glossary.

**Lesion (diameter)**	**Characteristics**
Macule (≤ 1 cm)<br>Patch (> 1cm)	Flat, circumscribed, non-palpable lesion that differs in color from the surrounding skin, of any color or shape
Papule (≤ 1 cm)	Elevated, solid, palpable lesion
Plaque (>1 cm)	Circumscribed, palpable lesion (can be elevated and can be secondary to a coalescence of papules)
Vesicle (≤ 1 cm)<br>Bulla (> 1cm)	Circumscribed lesion containing liquid (clear, serous or hemorrhagic)
Nodule (> 1 cm)	Elevated, solid lesion (i.e. no liquid contained)
Erosion	Loss of either a portion of or the entire epithelium (can be secondary to the detachment of the roof of a vesicle)
Ulcer	Full-thickness loss of the epithelium plus at least a portion of the underlying connective tissue

Supplementary Data 1. Initial research equation, refined research equation and full review references

("mouth mucosa"[MeSH Terms] OR "oral manifestations"[MeSH Terms] OR "tongue"[MeSH Terms] OR "cheek"[MeSH Terms] OR "lip"[MeSH Terms] OR "gingiva"[MeSH Terms] OR "palate"[MeSH Terms] OR "mucous membrane"[MeSH Terms] OR "mouth"[MeSH Terms]) AND ("stomatitis, herpetic"[MeSH Terms] OR "herpes labialis"[MeSH Terms] OR "chickenpox"[MeSH Terms] OR "herpes zoster"[MeSH Terms] OR "varicella zoster virus infection"[MeSH Terms] OR "infectious mononucleosis"[MeSH Terms] OR "cytomegalovirus infections"[MeSH Terms] OR "exanthema subitum"[MeSH Terms] OR "sarcoma, kaposi"[MeSH Terms] OR "herpangina"[MeSH Terms] OR "hand, foot and mouth disease"[MeSH Terms] OR "measles"[MeSH Terms] OR "mumps"[MeSH Terms] OR "papilloma"[MeSH Terms] OR "focal epithelial hyperplasia"[MeSH Terms] OR "condylomata acuminata"[MeSH Terms] OR "rubella"[MeSH Terms] OR "Chikungunya Fever"[Mesh Terms] OR "Dengue"[Mesh Terms] OR "hiv infections"[MeSH Terms] OR "molluscum contagiosum"[MeSH Terms] OR "erythema infectiosum"[MeSH Terms] OR "Parasitic Diseases"[Mesh Terms] OR "Virus Diseases"[Mesh Terms] OR "impetigo"[MeSH Terms] OR "scarlet fever"[MeSH Terms] OR "tonsillitis"[MeSH Terms] OR "actinomycosis"[MeSH Terms] OR "noma"[MeSH Terms] OR "tuberculosis, oral"[MeSH Terms] OR "tuberculosis"[MeSH Terms] OR "leprosy"[MeSH Terms] OR "pneumonia, bacterial"[MeSH Terms] OR "erythema multiforme "[MeSH Terms] OR "syphilis"[MeSH Terms] OR "chancre"[MeSH Terms] OR "candidiasis, oral"[MeSH Terms] OR "paracoccidioidomycosis"[MeSH Terms] OR "histoplasmosis"[MeSH Terms] OR "mucormycosis"[MeSH Terms] OR "aspergillosis"[MeSH Terms] OR "cryptococcosis"[MeSH Terms] OR "trichomonas infections"[MeSH Terms] OR "leishmaniasis"[MeSH Terms] OR "leishmaniasis, mucocutaneous"[MeSH Terms] OR “schistosomiasis"[MeSH Terms] OR "trypanosomiasis, african"[MeSH Terms] OR "Chagas disease"[MeSH Terms] OR "toxoplasmosis"[MeSH Terms] OR "cysticercosis"[MeSH Terms] OR "trichuriasis"[MeSH Terms] OR "echinococcosis"[MeSH Terms] OR "ascariasis "[MeSH Terms] OR "myiasis"[MeSH Terms])

[INITIAL RESEARCH EQUATION] NOT "stomatitis, herpetic"[MeSH Terms] NOT "herpes labialis"[MeSH Terms] NOT "chickenpox"[MeSH Terms] NOT "herpes zoster"[MeSH Terms] NOT "varicella zoster virus infection"[MeSH Terms] NOT "infectious mononucleosis"[MeSH Terms] NOT "herpangina"[MeSH Terms] NOT "hand, foot and mouth disease"[MeSH Terms] NOT "measles"[MeSH Terms] NOT "mumps"[MeSH Terms] NOT "papilloma"[MeSH Terms] NOT "focal epithelial hyperplasia"[MeSH Terms] NOT "condylomata acuminata"[MeSH Terms] NOT "hiv infections"[MeSH Terms] NOT "scarlet fever"[MeSH Terms] NOT "tuberculosis, oral"[MeSH Terms] NOT "tuberculosis"[MeSH Terms] NOT "pneumonia, bacterial"[MeSH Terms] NOT "erythema multiforme "[MeSH Terms] NOT "leishmaniasis"[MeSH Terms] NOT "cysticercosis"[MeSH Terms] NOT "candidiasis, oral"[MeSH Terms] NOT "paracoccidioidomycosis"[MeSH Terms] NOT "histoplasmosis"[MeSH Terms] NOT "mucormycosis"[MeSH Terms]

1. Araújo JF de, Oliveira AEF de, Carvalho HLCC de, Roma FRV de O, Lopes FF. Most common oral manifestations in pediatric patients HIV positive and the effect of highly active antiretroviral therapy. *Cienc Saude Coletiva*. 2018;23(1):115-122.

2. Orenuga OO, A O, Oluwakuyide RT, Olawuyi AB. Recurrent oral squamous papilloma in a pediatric patient: Case report and review of the literature. *Niger J Clin Pract*. 2018;21(12):1674-1677.

3. Ashton R, Wong KW, Weinstein M. Pediatric Lip Adhesion Following Bullous Erythema Multiforme and Review of Similar Oral Complications. *J Cutan Med Surg*. 2018;22(4):427-430.

4. Mohammadpour I, Motazedian MH, Handjani F, Hatam GR. Lip leishmaniasis: a case series with molecular identification and literature review. *BMC Infect Dis*. 2017;17(1):96.

5. Pinheiro R dos S, de França TRT, Ferreira D de C, Ribeiro CMB, Leão JC, Castro GF. Human papillomavirus in the oral cavity of children. *J Oral Pathol Med Off Publ Int Assoc Oral Pathol Am Acad Oral Pathol*. 2011;40(2):121-126.

6. Marques SA. Paracoccidioidomycosis: epidemiological, clinical, diagnostic and treatment up-dating. *An Bras Dermatol*. 2013;88(5):700.

7. Bicalho RN, Santo MF, de Aguiar MC, Santos VR. Oral paracoccidioidomycosis: a retrospective study of 62 Brazilian patients. *Oral Dis*. 2001;7(1):56-60.

8. Rioboo-Crespo M del R, Planells-del Pozo P, Rioboo-García R. Epidemiology of the most common oral mucosal diseases in children. *Med Oral Patol Oral Cirugia Bucal*. 2005;10(5):376-387.

9. Saint-Jean M, Tessier MH, Barbarot S, Billet J, Stalder JF, Société Française de Dermatologie Pédiatrique (SFDP). Oral disease in children. *Ann Dermatol Venereol*. 2010;137(12):823-837.

10. Pinto A, Haberland CM, Baker S. Pediatric soft tissue oral lesions. *Dent Clin North Am*. 2014;58(2):437-453.

11. Dilley DC, Siegel MA, Budnick S. Diagnosing and treating common oral pathologies. *Pediatr Clin North Am*. 1991;38(5):1227-1264.

12. Cleary KR, Batsakis JG. Mycobacterial disease of the head and neck: current perspective. *Ann Otol Rhinol Laryngol*. 1995;104(10 Pt 1):830-833.

13. Dilley DH, Blozis GG. Common oral lesions and oral manifestations of systemic illnesses and therapies. *Pediatr Clin North Am*. 1982;29(3):585-611.

14. Al Akhrass F, Debiane L, Abdallah L, et al. Palatal mucormycosis in patients with hematologic malignancy and stem cell transplantation. *Med Mycol*. 2011;49(4):400-405.

15. Romero de Leon E, Aguirre A. Oral cysticercosis. *Oral Surg Oral Med Oral Pathol Oral Radiol Endod*. 1995;79(5):572-577.

16. de Souza PE, Barreto DC, Fonseca LM, de Paula AM, Silva EC, Gomez RS. Cysticercosis of the oral cavity: report of seven cases. *Oral Dis*. 2000;6(4):253-255.

17. Saran RK, Rattan V, Rajwanshi A, Nijkawan R, Gupta SK. Cysticercosis of the oral cavity: report of five cases and a review of literature. *Int J Paediatr Dent*. 1998;8(4):273-278.

18. Vestey JP, Norval M. Mucocutaneous infections with herpes simplex virus and their management. *Clin Exp Dermatol*. 1992;17(4):221-237.

19. Vander Straten M, Tyring SK. Mucocutaneous manifestations of viral diseases in children. *Clin Dermatol*. 2002;20(1):67-73.

20. Myer C, Cotton RT. Salivary gland disease in children: a review. Part 1: Acquired non-neoplastic disease. *Clin Pediatr (Phila)*. 1986;25(6):314-322.

21. Felices RR, Ogbureke KU. Oral myiasis: report of case and review of management. *J Oral Maxillofac Surg Off J Am Assoc Oral Maxillofac Surg*. 1996;54(2):219-220.

22. Cohen L. Ulcerative lesions of the oral cavity. *Int J Dermatol*. 1980;19(7):362-374.

23. Bradley PJ. Benign salivary gland disease. *Hosp Med Lond Engl 1998*. 2001;62(7):392-395.

24. Miller GD, Tindall JP. Hand-foot-and-mouth disease. *JAMA*. 1968;203(10):827-830.

25. Strieder LR, León JE, Carvalho YR, Kaminagakura E. Oral syphilis: report of three cases and characterization of the inflammatory cells. *Ann Diagn Pathol*. 2015;19(2):76-80.

26. de Abreu MAMM, Alchorne MM de A, Michalany NS, Weckx LLM, Pimentel DRN, Hirata CHW. The oral mucosa in paucibacillary leprosy: a clinical and histopathological study. *Oral Surg Oral Med Oral Pathol Oral Radiol Endod*. 2007;103(5):48-52.

27. Kumar P, Savant SS. Solitary molluscum contagiosum. *Indian Pediatr*. 2015;52(8):723.

28. Crighton BK, Thomas C. Primary Ebstein-Barr Virus Infection Presenting with Prominent Lip and Tongue Swelling. *J Paediatr Child Health*. 2018;54(9):1052-1053.

29. Tungotyo M. Noma as a complication of false teeth (Ebiino) extraction: a case report. *J Med Case Reports*. 2017;11(1):112.

30. Raimondi F, Veropalumbo C, Coppola C, et al. Noma Neonatorum From Multidrug-Resistant Pseudomonas aeruginosa: An Underestimated Threat? *J Pediatr Infect Dis Soc*. 2015;4(3):25-27.

31. Zhang RZ, Jin HL. Syphilis in an Infant Acquired by Mouth-to-Mouth Transfer of Prechewed Food. *Pediatr Dermatol*. 2016;33(6):344-345.

32. Sun A, Chang JG, Chu CT, Liu BY, Yuan JH, Chiang CP. Preliminary evidence for an association of Epstein-Barr virus with pre-ulcerative oral lesions in patients with recurrent aphthous ulcers or Behçet's disease. *J Oral Pathol Med Off Publ Int Assoc Oral Pathol Am Acad Oral Pathol*. 1998;27(4):168-175.

33. de Carvalho CHP, de Andrade ALDL, de Oliveira DHIP, Lima E das N de A, da Silveira EJD, de Medeiros AMC. Intraoral molluscum contagiosum in a young immunocompetent patient. *Oral Surg Oral Med Oral Pathol Oral Radiol*. 2012;114(1):57-60.

34. de Araújo RJG, Corrêa AM, Santos WRA, Moreira MT. Advanced stage of oral myiasis in children: a clinical case report. *Quintessence Int Berl Ger 1985*. 2008;39(1):39-43.

35. Yamamoto K. Oral Manifestation Like Forchheimer Spots of Dengue Fever. *Am J Trop Med Hyg*. 2019;101(4):729.

36. Costa Paiva SS, Viana de Siqueira AP, Damaceno Costa L, Saddi Godinho TC, Marques Fortes P, Sucasas Costa P. Invasive oral and nasal aspergillosis in an immunocompetent child. *Lancet Infect Dis*. 2020;20(2):260.

37. Sharma A, Hedge A. Primary oral myiasis due to Chrysomya bezziana treated with Ivermectin. A case report. *J Clin Pediatr Dent*. 2010;34(3):259-261.

38. Karabulut AB, Kabakas F, Berköz O, Karakas Z, Kesim SN. Hard palate perforation due to invasive aspergillosis in a patient with acute lymphoblastic leukemia. *Int J Pediatr Otorhinolaryngol*. 2005;69(10):1395-1398.

39. Dhanrajani A, Khubchandani RP. Acute gangrenous gingivitis - early stage noma. *Indian Pediatr*. 2014;51(4):333.

40. Andrade-Filho J de S. Analogies in medicine : slapped cheek appearance. *Rev Inst Med Trop São Paulo*. 2014;56(5):458.

41. Chua KB, Lam SK, AbuBakar S, et al. The predictive value of uvulo-palatoglossal junctional ulcers as an early clinical sign of exanthem subitum due to human herpesvirus 6. *J Clin Virol Off Publ Pan Am Soc Clin Virol*. 2000;17(2):83-90.

42. el Mostehy MR, Stallard RE. Oral manifestations in Egyptian hepatosplenic bilharziasis. *J Periodontol*. 1968;39(4):236-240.

43. Menni S. Acquired primary syphilis on a child's lip. *Acta Derm Venereol*. 2007;87(3):284.

44. Saez J, Pinto P, Apt W, Zulantay I. Cystic echinococcosis of the tongue leading to diagnosis of multiple localizations. *Am J Trop Med Hyg*. 2001;65(4):338-340.

45. Freeman AF, Mancini AJ, Yogev R. Is noma neonatorum a presentation of ecthyma gangrenosum in the newborn? *Pediatr Infect Dis J*. 2002;21(1):83-85.

46. Chua KB, Lam SK, Sazaly AB, Lim ST, Paranjothy M. Uvulo-palatoglossal junctional ulcers--an early clinical sign of exanthem subitum due to human herpesvirus 6. *Med J Malaysia*. 1999;54(1):32-36.

47. Tempest MN. Cancrum oris. *Br J Surg*. 1966;53(11):949-969.

48. Chua KB. The association of uvulo-palatoglossal junctional ulcers with exanthem subitum: a 10-year paediatric outpatient study. *Med J Malaysia*. 1999;54(1):58-64.

49. Roveda SI. Cervicofacial actinomycosis. Report of two cases involving major salivary glands. *Aust Dent J*. 1973;18(1):7-9.

50. Whitefield S, Raiser V, Shuster A, Kleinman S, Shlomi B, Kaplan I. The Spectrum of Oral Lesions Presenting Clinically With Papillary-Verrucous Features. *J Oral Maxillofac Surg Off J Am Assoc Oral Maxillofac Surg*. 2018;76(3):545-552.

51. Lipner SR, Scher RK. Periungual and Oral Verruca. *J Cutan Med Surg*. 2017;21(3):251.

52. Sanmark E, Wikstén J, Välimaa H, Aaltonen LM, Ilmarinen T, Blomgren K. Peritonsillar abscess may not always be a complication of acute tonsillitis: A prospective cohort study. *PloS One*. 2020;15(4).

53. Gupta R, Gupta R, Sethi S, Khanal M. Isolated Unilateral Soft Palate Palsy Following Tonsillopharyngitis Caused by Epstein-Barr Virus Infection. *Cleft Palate-Craniofacial J Off Publ Am Cleft Palate-Craniofacial Assoc*. 2017;54(3):351-353.

54. Baldawa P, Shirol P, Kulkarni D, Koshti S, Mishra N. Actinomycotic osteomyelitis of palate masquerading periapical pathology: A rare case report. *Indian J Dent Res Off Publ Indian Soc Dent Res*. 2018;29(2):247-251.

55. Pontes FSC, Frances LTM, Carvalho M de V, et al. Severe oral manifestation of dengue viral infection: a rare clinical description. *Quintessence Int Berl Ger 1985*. 2014;45(2):151-156.

56. Summersgill KF, Smith EM, Levy BT, Allen JM, Haugen TH, Turek LP. Human papillomavirus in the oral cavities of children and adolescents. *Oral Surg Oral Med Oral Pathol Oral Radiol Endod*. 2001;91(1):62-69.

57. Bandyopadhyay D, Ghosh SK. Mucocutaneous features of Chikungunya fever: a study from an outbreak in West Bengal, India. *Int J Dermatol*. 2008;47(11):1148-1152.

58. Sun A, Chang JG, Kao CL, et al. Human cytomegalovirus as a potential etiologic agent in recurrent aphthous ulcers and Behçet's disease. *J Oral Pathol Med Off Publ Int Assoc Oral Pathol Am Acad Oral Pathol*. 1996;25(5):212-218.

59. Baratti-Mayer D, Pittet B, Montandon D. [GESNOMA (Geneva Study Group on Noma): state-of-the-art medical research for humanitarian purposes]. *Ann Chir Plast Esthet*. 2004;49(3):302-305.

60. Bon A, Eichmann A, Grob R. Focal epithelial hyperplasia. *Dermatol Basel Switz*. 1992;184(4):294-295.

61. Tempest MN. Cancrum oris. *Trop Doct*. 1971;1(4):164-169.

62. Walsh S. Stick out your tongue and say...uh, oh! *J Pediatr Health Care Off Publ Natl Assoc Pediatr Nurse Assoc Pract*. 2005;19(4):247-248, 263-264.

63. Morgado de Abreu MAM, Neto Pimentel DR, Morgado de Abreu NM, et al. Presence of oral lesions in leprosy patients does not imply involvement by Mycobacterium leprae. *Oral Surg Oral Med Oral Pathol Oral Radiol Endod*. 2010;109(3):328-329.

64. Horie Y, Kato M. Juvenile hemangioma (infantile hemangioendothelioma) of the parotid gland associated with cytomegalovirus infection. *Pathol Int*. 1999;49(7):668-671.

65. al-Ismaily M, Scully C. Oral myiasis: report of two cases. *Int J Paediatr Dent*. 1995;5(3):177-179.

66. Bouckaert MM, Raubenheimer EJ, Jacobs FJ. Maxillofacial hydatid cysts. *Oral Surg Oral Med Oral Pathol Oral Radiol Endod*. 2000;89(3):338-342.

67. Malbora B, Avci Z, Ozbek N. Buccal and gingival necrotic plaques in a 14-year-old patient. *Pediatr Dermatol*. 2012;29(4):519-520.

68. Feigin RD, Shearer WT. Opportunistic infection in children. III. In the normal host. *J Pediatr*. 1975;87(6 Pt 1):852-866.

69. Burgoon CF, Burgoon JS. Viral and rickettsial diseases of the skin and mucous membranes. *Pediatr Clin North Am*. Published online 1956:521-554.

70. Meyer PM, Streuli JC, Schlumbom V, Goetschel P. A girl with a purpuric rash (case presentation). *Acta Paediatr Oslo Nor 1992*. 2010;99(3):327-328, 475-476.

71. Ginsburg CM. Management of selected skin and soft tissue infections. *Pediatr Infect Dis*. 1986;5(6):735-740.

72. Mahajan VK, Sharma NL. Scarlet fever. *Indian Pediatr*. 2005;42(8):829-830.

73. Ataoglu H, Uckan S, Oz G, Altinörs N. Maxillofacial hydatid cyst. *J Oral Maxillofac Surg Off J Am Assoc Oral Maxillofac Surg*. 2002;60(4):454-456.

74. Murray MJ, Murray AB, Murray MB, Murray CJ. Parotid enlargement, forehead edema, and suppression of malaria as nutritional consequences of ascariasis. *Am J Clin Nutr*. 1977;30(12):2117-2121.

75. Antonio AG, Kelly A, Maia LC. Premature loss of primary teeth associated with congenital syphilis: a case report. *J Clin Pediatr Dent*. 2005;29(3):273-276.

76. Souza VA, Emmerich A, Coutinho EM, et al. Dental and oral condition in leprosy patients from Serra, Brazil. *Lepr Rev*. 2009;80(2):156-163.

77. Appel BN, Mendelow H, Pasqual HN. Acquired toxoplasma lymphadenitis. *Oral Surg Oral Med Oral Pathol*. 1979;47(6):529-532.

78. Barsh LI. Molluscum contagiosum of the oral mucosa. Report of a case. *Oral Surg Oral Med Oral Pathol*. 1966;22(1):42-46.

79. Williams HK, Edwards MB, Adekeye EO. Onchocerciasis of the face and mouth. *Oral Surg Oral Med Oral Pathol*. 1986;62(5):560-563.

80. Oudidi A, Benmansour N, El Alami EAMN. [Hydatid cyst of the parotid gland: six cases]. *J Otolaryngol*. 2006;35(5):317-319.

81. Reichart P. Pathologic changes in the soft palate in lepromatous leprosy. An evaluation of ten patients. *Oral Surg Oral Med Oral Pathol*. 1974;38(6):898-904.

82. Akiner MN, Saatci MR, Yilmaz O, Erekul S. Intraglandular toxoplasmosis lymphadenitis of the parotid gland. *J Laryngol Otol*. 1991;105(10):860-862.

83. Altman MM, Gutman D. Echinococcus of the parotid gland. *J Laryngol Otol*. 1966;80(4):409-411.

84. Kumar V, Singh AP, Meher R, Raj A. Primary Tuberculosis of Oral Cavity: A Rare Entity Revisited. *Indian J Pediatr*. 2011;78(3):354-356.

85. Arora VK, Chopra N, Singh P, Venugopal VK, Narang S. Hydatid cyst of parotid: Report of unusual cytological findings extending the cytomorphological spectrum. *Diagn Cytopathol*. 2016;44(9):770-773.

Supplementary Figure 1. Flow chart of the articles' selection process

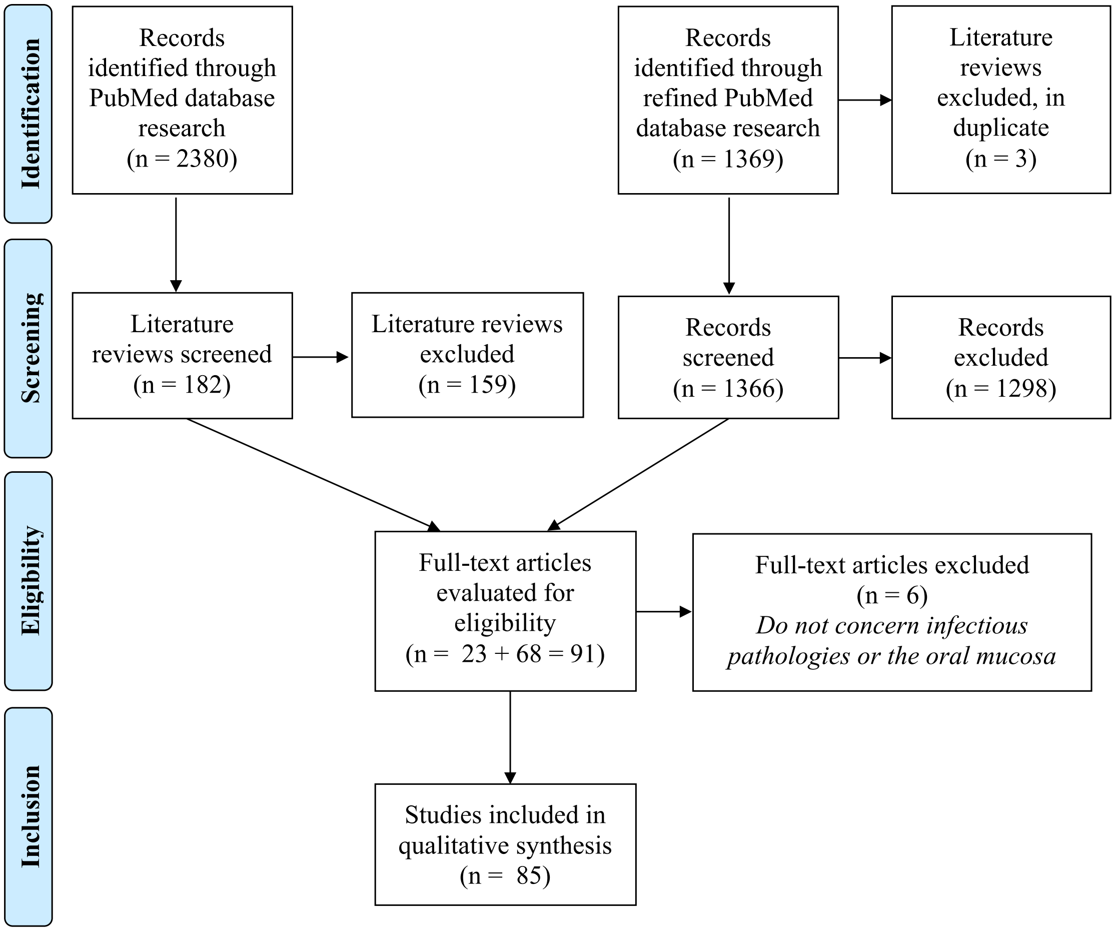

Supplement: Supplementary file 1 [file Table_1.pdf]
